# Depression and Anxiety Disorders in Patients With Inflammatory Bowel Disease

**DOI:** 10.3389/fpsyt.2021.714057

**Published:** 2021-10-08

**Authors:** Shurong Hu, Yiping Chen, Yan Chen, Caihua Wang

**Affiliations:** ^1^Department of Gastroenterology, The Second Affiliated Hospital, Zhejiang University School of Medicine, Hangzhou, China; ^2^Department of Psychiatry, The Second Affiliated Hospital, Zhejiang University School of Medicine, Hangzhou, China

**Keywords:** depression disorders, anxiety disorders, inflammatory bowel disease, psychological treatment, diagnose of psychological disorders

## Abstract

Mental health is a significant yet overlooked aspect of inflammatory bowel disease (IBD) patient care, with challenges in determining optimal treatments and psychological health resources. The most common psychological conditions in patients with IBD are anxiety and depression. The increased prevalence of these mental disorders appeals to mental screening of each person diagnosed with IBD at initial consultation. There are simple and clinically viable methods available to screen for mental problems. Psychological methods may be as or even more significant as a therapeutic modality. Herein we discuss the three major areas of psychological co-morbidity in IBD: (1) the prevalence and risk factors associated with anxiety and depression disorders for patients with IBD; (2) diagnosis of psychological disorders for patients with IBD; (3) treatment with patients with IBD and mental disorders. The gastroenterologists are encouraged to screen and treat these patients with IBD and mental disorders, which may improve outcomes.

## Introduction

Inflammatory Bowel Diseases (IBD), including Crohn's disease (CD) and ulcerative colitis (UC), are characterized as chronic and relapsing inflammatory disorders with the peak incidence around 20 years of age (1). The incidence and prevalence of IBD are increasing worldwide. In China, the incidence rate of IBD was 1.74 per 100,000 person-years (UC: 1.18 per 100,000; CD: 0.4 per 100,000) (1). The patients with IBD had significant physical, psychosocial, and economic burden related to high risk of adverse health outcomes, including morbidity, hospitalization, surgery, work disability and even mortality (2). Health-related quality of life (HRQoL) is a concept that includes domains related to physical factors (such as natural history of disease and medications), mental factors (such as anxiety and depression), and social factors (such as job status and occupations) (3). Anxiety and depression are the most common psychological disorders for patients with IBD. One recent study showed that patients with IBD and mental health disease generated significantly higher costs compared with patients without mental disorders (4). Psychological disorders in patients with IBD may lead to high risk of relapse and poor treatment compliance. Moreover, systematic literature review suggested that psychiatric treatment may be effective in reducing anxiety and depression disorders, gastrointestinal symptoms and disease activity (5).

It has long been believed that patients with IBD might have psychological illness, including symptoms of common mental disorders and somatization, which may be related to the complex bidirectional interaction via the gut-brain axis (6). In a study of 405 patients with IBD following for 2 years, the authors observed that patients with normal anxiety scores but who were active at baseline had a nearly 6-fold increase in the risk of anxiety symptoms (7). Therefore, the occurrence of anxiety and depression may be related to the manifestations of active intestinal inflammation related to brain-gut axis imbalance (6, 7).

The identified articles for this review used three different electronic databases: MEDLINE, PsychINFO, and EMBASE. The search terms were used: “inflammatory bowel diseases”, “ulcerative colitis”, “Crohn's disease”. The set operator AND was used to combine these terms with the following terms: “anxiety”, “depression”, and “mood disorder” or “mental disorder”. Herein we discuss the three major areas of psychological co-morbidity in IBD: (1) the prevalence and risk factors associated with anxiety and depression disorders for patients with IBD; (2) diagnosis of psychological disorders for patients with IBD; (3) treatment with patients with IBD and mental disorders. The clinicians are encouraged to detect and treat these patients with IBD and mental disorders, which may improve outcomes.

### Prevalence and Risk factors Associated With Anxiety and Depression Disorders

Compared with the general population, patients with IBD had a higher lifetime rate of psychiatric disorders such as anxiety and depression, which were known to have negative impact on the quality of their lives and the severity of disease (8, 9). Recently, in a systemic review and meta-analysis, the authors performed subgroup analyses by gender, disease location and disease activity, country and method used to define anxiety and depression (10). The meta-analysis showed that patients with IBD were exposed to high prevalence of symptoms of anxiety and depression, with approximately one in three patients affected by anxiety symptoms and one in four patients affected by depression symptoms (10). Furthermore, the prevalence of symptoms of anxiety or depression were higher inpatients with active IBD than in patients with inactive disease (10). Another study reported that patients with IBD had 3–5 times more likely to develop anxiety disorders and 2–4 times to develop depression disorders in the lifetime compared with general population (11).There was a Korea nationwide study with 46,707 non-IBD as control and 15,569 patients with IBD who were followed for 6 years, patients with IBD experienced significantly more anxiety (12.2 vs. 8.7%) and depression (8.0 vs. 4.7%) (12). One systematic review showed that patients with IBD had ~15 and 20%prevalence rate of depression and anxiety respectively (13). It was worth mentioning that one population-based study demonstrated that depression and anxiety disorders might precede IBD diagnosis by at least 5 years (14). In another population-based cohort study in Canada, nearly 80% of patients with IBD and anxiety were diagnosed with anxiety more than 2 years before the diagnosis of IBD (11).

By identifying factors related to the development of mental disorders in patients with IBD, it is possible to reverse anxiety and depressive disorders, not only to improve the quality of life, but also to improve the course of the disease. Based on data from the ISSEO cohort studies, factors associated with anxiety and depression disorders were severe disease, disease flares and socioeconomic deprivation (15). The Korea national study demonstrated a higher risk of new onset anxiety among individuals with CD (hazard ratio, 1.63) or UC (hazard ratio, 1.60) compared with matched controls. The risk ratios of depression after diagnosis of CD and UC are 2.09 and 2.00, respectively (12). This phenomenon was also observed in a case-control study in southern England, indicating that the risk of anxiety and depression is highest within 1 year after IBD diagnosis (16). Another prospective longitudinal follow-up conducted in Ontario, Canada showed that patients with abnormal anxiety HADS (Hospital Anxiety and Depression Scale, HADS) scores had higher IBD-related outcomes compared with patients whose anxiety scores did not increase (odds ratio, 3.36) (17). However, no difference was observed in those with abnormal depression HADS scores compared with those without elevated depression scores (odds ratio, 0.43) in IBD-related outcomes (17). Moreover, the results suggested that elevated anxiety HADS scores at baseline was an independent predictor of adverse IBD-related outcomes (17). A study of 204 patients with IBD (103 with UC and 101 with CD) showed that perceived stress was associated with mood disorders in IBD (18). However, in UC, other factors like a new diagnosis of IBD were related to anxiety and stress, while inpatient status and active diseases were related to depression. In CD, the factors such as abdominal pain, lower socioeconomic status were related to anxiety, while increasing age was related to depression (18).

Furthermore, large longitudinal studies had consistently shown more frequent IBD flares and worse disease activity in those with symptoms of anxiety and depression disorders (19). In a cohort of 2,007 patients followed over 9 years from the Swiss, the results showed an association between depressive symptoms and clinical recurrence of IBD (20). In a large longitudinal study of patients with IBD (1,516 with UC and 2,798 with CD) from USA with almost 2 years follow-up, Bharati Kochar et al. found that baseline depression patients with CD have an increased risk of recurrence (risk ratio, RR: 2.3), hospitalization (RR:1.3) or IBD-related surgery (RR:1.3). UC patients with baseline depression also have an increased risk of relapse (RR: 1.6), hospitalization (RR: 1.3), or surgery (RR: 1.8) at follow-up (21).

In conclusion, new onset of disease, disease activity, side effects of medications, stressful life events, inpatient status and lower socioeconomic status can easily affect the mood of one or two types of IBD (22). The increased prevalence of anxiety and depression disorders required a mental screening of each patient with IBD at initial consultation.

### Diagnosis of Psychological Disorders

Identifying the psychological disorders of patients with IBD at the time of diagnosis or during the course of the disease is highly related to the patient's care, timely initiation of appropriate treatment, or improvement of the outcome of the disease (23, 24). Screening and monitoring the psychological disorders in patients with IBD play significant role in both primary care and specialist settings. Unfortunately, no IBD specific instruments to measure anxiety or depression disorders have been validated to date. Tools developed for common illnesses of depression and anxiety include self-reported or clinician-reported approaches, and structured clinician psychiatric interviews (25). One approach to determine anxiety and depression is to use existing screening measures that have established reliability and effectiveness, which are easy to score and can be coded into electronic medical records. No measure can replace the doctor's judgment, and all positive screenings should be linked to an action plan involving further patient evaluation and treatment (26). It was important that clinicians and researchers understood the strengths and weaknesses of the various screening tools, and were informed about the interpretation of the resultant scores. The questionnaire scales and cutoff used to define presence of patients with symptoms of anxiety or depression were different (10). The detailed characteristics of depression and anxiety symptom scales were described as follows (also listed in Table 1).

**Table 1:** A summary of the characteristics of the scales.

**Scale**	**Items (*n*)**	**Total score**	**Cut-point**	**Sensitivity (%)**	**Specificity (%)**
**Anxiety**					
HADS-A	7	21	8	90.0	78.0
PROMIS-A	8	T 38-81	T score 60	86.0	81.6
HAM-A	14	56	17	85.7	63.5
GAD-7	7	21	>10	89.0	82.0
**Depression**					
HADS-D	7	21	8	80.0	69.0
PROMIS-D	8	T 38-81	T score 60	82.3	81.4
HAM-D	17	56	20	86.4	92.2
PHQ-9	9	0–27	10	83.0	86.0
PHQ-2	2	0–6	3	97.9	67.0
WBI-5	5	25	<12	61.0	92.0

#### Structured Clinical Interview for DSM Disorders (SCID)

The SCID is a semi-structured clinical interview that uses DSM-IV (Diagnostic and Statistical Manual of Mental Disorders IV criteria) to identify mental illness. SCID-based diagnosis can be used as a reference standard for standard validity analysis (14). “SCID depression” refers to any current or lifetime SCID diagnosis of major depression or thymic disorder; “SCID anxiety disorder” refers to any current or lifetime SCID diagnosis of panic disorder (27).

#### Hospital Anxiety and Depression Scale (HADS)

The HADS is a 14-item self-report questionnaire used to assess anxiety (A) and depression (D) levels in the past week (28). Each question is assessed using a 4-point like rt scale and the total score is 42 (21 with depression and 21 with anxiety). And the higher scores, the larger degree of anxiety and depression. It has been extensively studied to describe the prevalence of anxiety and depression in IBD developed for outpatients. The items on the scale are less directly influenced by disease-related symptoms which are different from other scales (18, 29). The cut-point of 8 is used to identify possible anxiety or depression (18, 29). The detection sensitivity and specificity of HADS are 80 and 69% for depression, respectively, and 90 and 78% for anxiety (28). However, HADS-D and HADS-A are the lowest sensitivity relative to other measures.

#### Patient-Reported Outcomes Measurement Information System (PROMIS)

The PROMIS, including Depression Short-Form 8a (PROMIS Depression) and Anxiety Short-Form 8a (PROMIS Anxiety), is an 8-item self-report questionnaire used to assess the level of anxiety and depression in the past week (30). The American Psychiatric Association has provided guidance on the use of a native scale for PROMIS Anxiety and Depression to identify the severity of psychological distress (31, 32). Among other outcome variables, the PROMIS instrument generates anxiety and depression scores (33). The raw scores for anxiety and depression are translated to standardized T-scores with a population mean of 50 ± 10 (30, 34). Unlike other measures, the PROMIS instruments are based on the item response theory framework to develop item banks, which allow delivery by computer-adaptive testing (33). PROMIS anxiety and depression can detect generalized anxiety screening positive (sensitivity 86.0%; specificity 81.6%) and possible major depression (sensitivity 82.3%; specificity 81.4%) (35). However, scoring PROMIS response patterns for research purposes can be very time-consuming.

#### Hamilton Rating Scale (HAM)

Hamilton Anxiety Rating Scale (HAM-A) and Hamilton Depression Rating Scale (HAM-D) are the **two** oldest tools for assessing anxiety and depression (36, 37). These are scales assessed by clinicians and consist of 14 and 17 items respectively. They are used to assess the severity of patient anxiety and depression, which are the most used anxiety and depression scale assessed by clinicians in the world. Interestingly, the main value of HAM-A and HAM-D is to assess the patient's response to a course of treatment, not a diagnostic or screening tool (39). Through continuous use of the scale, doctors can evaluate the results of medication or psychotherapy (38). The detection sensitivity and specificity of HAM-A are 85.7 and 63.5% for anxiety, and 86.4 and 92.2% for depression, respectively.

#### Patient Health Questionnaire-9 (PHQ-9)

The PHQ-9 is a self-administered scale developed by Spitzer et al. for screening depression which is freely available in more than 60 languages (39). The shorter versions without the suicidal item are PHQ-4 and PHQ-2. The total score is 27 and the cut off score for PHQ-9 is 10 (40). The management time of the questionnaire is <5 min. The PHQ scale is a multi-purpose instrument that can also be used to diagnose, monitor and measure the severity of depression (39). The sensitivity and specificity of this scale are 83 and 86%, respectively (41). While the detection sensitivity and specificity of PHQ-2 are 97 and 67% for depression, respectively (42).

#### World Health Organization Well-Being index (WBI-5)

The WBI-5 is a short questionnaire consisting of five simple non-invasive questions.

It has sufficient effectiveness in screening depression and measuring the results of clinical trials, and has been successfully applied in a wide range of research fields (43). Currently, WBI-5 has been translated into more than 30 languages and has been used in research projects around the world. The total score is 25, and score <12 is consistent with clinical depression (44). Moreover, the WBI-5 detects depression with 61% insensitivity and 92% in specificity (43).

#### Symptom Checklist Anxiety Scale (SCL-A20)

SCL-A20 is a subscale of Symptom Checklist 90 Revised (SCL90-R) designed by Derogatis LR, which composed of 20 items and is used as a screening tool for anxiety (45). SCL90-R is composed of 90 items and assesses a wide range of mental symptoms, which have been widely used and proven to be reliable. Compared to SCL90-R, SCL-A20 takes a shorter time to administer. The cut off score of SCL-A20 for clinical anxiety is 30.

#### Generalized Anxiety Disorder-7 (GAD-7)

The GAD-7 is a 7-item self-report questionnaire assessing levels of anxiety over the last 2 weeks, which has demonstrated promise as a scale with good clinical applicability and powerful psychometric characteristics (46). It has demonstrated good reliability and structural validity (47, 48). The total score is 21, and score >10 is consistent with moderate anxiety. Furthermore, GAD-7 has a sensitivity of 89% for GAD and a specificity of 82%, which moderately good at screening panic disorder (sensitivity 74%, specificity 81%), social anxiety disorder (sensitivity 72%, specificity 80%) and post-traumatic stress disorder (sensitivity 66%, specificity 81%), etc., (47, 49). However, GAD-7 may not be able to capture the anxiety associated with anxiety disorders other than generalized anxiety disorder.

Moreover, a study systematically assessed the reliability and validity of multiple symptom rating scales for anxiety and depression in patients with IBD (14). The study showed that the symptom rating scales for anxiety and depression were similar in psychometric attributes. However, the anxiety scale was not as good as the depression scale (14). To be mentioned, most studies on self-reported symptom scales that assess anxiety and depression are conducted in primary care settings, but not in people with chronic diseases (such as IBD) who suffer from depression. The risk of illness and anxiety is particularly high, and the result may be higher (50). However, the accurate screening rate for mental disorders in primary care is estimated to be <50%, and the recognition rate for depression in primary care may be only 3.4% (51–53). Future research will need to determine to what extent the consistent use of the symptom scale in the IBD population will improve the detection of depression and anxiety. Clinicians evaluating patients with IBD should incorporate symptom scales into their practice, provided that when the score exceeds a certain cut-off point for the population, appropriate follow-up actions are available to clarify the background and manage symptoms. Unless we have more precise and standardized tools to quantify the psychological and inflammatory aspects of IBD, the dispute on the link between common mental disorders and IBD may continue. Therefore, clinicians may improve their detection of depression and anxiety through carrying out a symptom scale.

### Treatment

Supporting care that includes medical care and psychosocial, environmental, and behavioral interventions to improve health, which may achieve the highest health value (54). Both anxiety disorders and depression can be treated effectively by pharmacotherapy as well as psychotherapy (Table 2). Psychological therapies should be considered in patients with IBD and functional symptoms (55). Most cases of mental disorders can be successfully treated with behavioral interventions, such as cognitive behavioral therapy (CBT), hypnosis, and mindfulness techniques for patients with and without IBD (56–60). In CBT, CBT has not been studied to continuously change the outcome of the disease, but it can effectively improve medical compliance and underlying symptoms of anxiety or depression (61, 62). CBT is better than usual controls and treatments, but not better than other positive behavioral interventions. Mindfulness meditation programs can regulate pain, anxiety and depression of various physical health diseases (63). Hypnotherapy is a promising adjunctive treatment for IBD and has further proved beneficial for pain and anxiety in non-IBD populations (64, 65). One study demonstrated the effect of 12 hypnotherapy on patients with IBD taking steroids for 5 years, the result showed that 60% patients stopped using steroids after hypnotherapy (66). Although hypnosis is efficacious, there are few behavioral professionals trained in medical hypnosis.

**Table 2:** The behavioral interventions and psychopharmacology treatment.

	**Types of behavioral interventions**	**Psychopharmacology**
Anxiety	CBT	SSRI
	Hypnosis	SNRI
	Mindfulness	Mirtazapine
	Meditation	Buspirone
Depression	CBT	SSRI
	Mindfulness	SNRI
		Mirtazapine
		Buspirone

Along with psychotherapy, antidepressants have been recognized as treatments for gut–brain disorders that might benefit both psychological and gastrointestinal health (67). Antidepressants have been found to be effective for the treatment of depression, anxiety, and chronic pain syndromes, but the overall support for their efficacy is modest at best. Although there are no large randomized trials for patients with IBD, there is evidence that selective serotonin reuptake inhibitors (SSRIs), serotonin and norepinephrine reuptake inhibitors (SNRIs), and tricyclic antidepressants (TCAs) can reduce anxiety and depression psychotherapy when used in combination with the following drugs (68–70).

#### SSRIs

Several clinical trials have addressed the impact of SSRI treatment on gastrointestinal dysfunction (71). The impact of SSRI on IBD is controversial. Most studies have shown that SSRI treatment has some benefits for patients with IBD, while other studies have failed to prove superior to placebo, especially if patients with depression are excluded from the trial (72, 73). SSRI antidepressants can help relieve symptoms of depression, such as depression, irritability, feelings of worthlessness, irritability, anxiety, and trouble sleeping (74). SSRIs are generally well tolerated. However, like most antidepressants, SSRIs are associated with activation and increased suicidal ideation at higher doses.

#### SNRIs

SNRIs are a newer category that can block presynaptic serotonin and norepinephrine transporters, thereby increasing the post-synaptic stimulation of these receptors, which may be useful for refractory depression, anxiety, and chronic pain. The symptom is effective, and its side effects are similar to SSRIs and TCAs, with minor side effects (75).

#### TCAs

Although TCA may have many serious side effects, TCA has shown some efficacy on functional gastrointestinal symptoms. In the Australian IBD treatment survey, approximately one-third of patients used antidepressants including TCA (69). The most common reason for this prescription is depression or anxiety, but TCA is rarely used for physical illness. A second retrospective study found that high-dose TCA prescribed for depression can reduce disease episodes, endoscopy and steroid use (76).

#### Mirtazapine

Mirtazapine is a noradrenergic and specific serotonergic antidepressant (NaSSA) with a variety of unique receptor activities, including blocking postsynaptic 5-HT2 and 5-HT3 receptors, Inhibition of presynaptic α2-adrenergic receptors, potent histamine H1 antagonism and moderate muscarinic receptor antagonism. The patients who did not tolerate SSRI therapy had efficacyfrom50 to 73% when treated with Mirtazapine (77). A study has shown that it is effective in the treatment of IBS, which is dominated by diarrhea and accompanied by depression and anxiety (78). If the side effects of sedation and weight gain can be tolerated, mirtazapine has a moderate effect on depression, and there is some support for the use of nausea and vomiting (68).

#### Bupropion

Compared with SSRIs, bupropion seems to be more effective in treating drowsiness and fatigue, but has less benefit in depression-related anxiety (73, 79). Bupropion targets the reuptake of dopamine and norepinephrine, has a moderate effect on depression, and has a certain supportive effect on fatigue and smoking cessation (68, 80). Buspirone has indications for generalized anxiety disorder, but studies have shown only small benefits. There is currently more and more evidence to treat functional dyspepsia (68). Unlike benzodiazepines, it has no side effects of physical dependence and cognitive impairment (81, 82).

Most of these drugs have physiological effects on the brain, immune system and gastroenterology (except bupropion), so their treatment and side effects are represented in these systems. In a national study in Denmark, it was found that the use of antidepressants is beneficial to the course of patients with UC and CD, especially those who did not use antidepressants before the onset of IBD (83). A systematic review showed that antidepressant treatment may have a beneficial effect on IBD activity, disease recurrence, IBD-related surgery, quality of life, and treatment compliance (5, 83–85). In the comparison of IBD depression patients who insisted and did not adhere to antidepressant treatment, the IBD activity, depression and anxiety of the patients who insisted on treatment decreased, and the quality of life improved (8). Some studies have shown that patients with IBD are more prone to depression, which can worsen the prognosis of IBD (86). Recently, an analysis of a large retrospective cohort study showed that patients with depression have a significantly higher risk of CD (adjusted HR: 2.11) and UC (adjusted HR: 2.23) (85). In addition, this study showed that SSRIs and TCAs have a protective effect on CD, while mirtazapine, SNRIs, SSRIs, serotonin modulators and TCAs have a protective effect on UC (85). The study showed that depression increases the risk of IBD, and the use of antidepressants during the treatment of depression may reduce this risk.

### Conclusions and Perspectives

Psychological conditions like anxiety and depression are common and can affect the quality of life and the course of IBD. The increasing prevalence of these mental illnesses requires that every person diagnosed with IBD be mentally screened during the initial consultation. Therefore, it is necessary and feasible to call for patients with IBD to see a psychologist once a year. There are simple and clinically feasible tools that can be used to screen for psychological problems. Psychological factors are essential for proper IBD management, as there are complex interactions between the physical and emotional health of patients with IBD. Psychological methods may be as important as treatment methods, or even more important. Behavioral interventions can improve outcomes in part by changing relationships, compliance, and preventing recurrence. Antidepressants may have a beneficial effect on IBD activity, disease recurrence, IBD-related surgery, quality of life, and treatment compliance. The comprehensive management method of patients with IBD composed of gastroenterologists and psychiatrists should be included as the standard of care.

## Author Contributions

CW and YaC: conceptualization. SH: writing - original draft. YiC and CW: writing - review and editing. All authors contributed to the article and approved the submitted version.

## Conflict of Interest

The authors declare that the research was conducted in the absence of any commercial or financial relationships that could be construed as a potential conflict of interest.

## Publisher's Note

All claims expressed in this article are solely those of the authors and do not necessarily represent those of their affiliated organizations, or those of the publisher, the editors and the reviewers. Any product that may be evaluated in this article, or claim that may be made by its manufacturer, is not guaranteed or endorsed by the publisher.
